# *Woodfordia fruticosa* extract nanoemulsion: Influence of processing treatment on droplet size and its assessment for *in vitro* antimicrobial and anti-inflammatory activity

**DOI:** 10.3389/fnut.2022.944856

**Published:** 2022-09-26

**Authors:** Agnieszka Najda, Aarti Bains, Joanna Klepacka, Prince Chawla

**Affiliations:** ^1^Department of Vegetable and Herbal Crops, The University of Life Science in Lublin, Lublin, Poland; ^2^Department of Microbiology, Lovely Professional University, Phagwara, India; ^3^Department of Commodity Science and Food Analysis, Faculty of Food Science, University of Warmia and Mazury in Olsztyn, Olsztyn, Poland; ^4^Department of Food Technology and Nutrition, Lovely Professional University, Phagwara, India

**Keywords:** Lythraceae, nanoemulsion (nanoE), antioxidant, anti-inflammatory, zeta potential (ZP), gum arabic

## Abstract

Recently, plant-derived bioactive compounds have been utilized in the preparation of several functional food products; however, stability and water solubility are major constraints to these compounds. Therefore, to overcome this problem, the synthesis of nanoemulsion (oil in water) with varying concentrations of *Woodfordia fruticosa* flower extract (1%−10% w/v) was carried out and characterization of nanoemulsion was done. The average droplet size of nanoemulsion samples ranges from 149.25 to 244.33 nm. The control and WFNE3 nanoemulsion showed significantly (*p* < 0.05) higher thermal stability when correlated with average droplet size. An insignificant difference (*p* > 0.05) was observed in the average droplet size and zeta potential WFNE3 (−30.3mV) with the increased temperature rate. At varied pH ranges, WFNE3 showed significantly higher (*p* < 0.05) stability in comparison with the control nanoemulsion sample. In terms of ionic strength, WFNE3 nanoemulsion sample showed significantly (*p* < 0.05) higher stability, and with an increasing concentration of salt, the colloidal system of the WFNE3 sample showed significantly (*p* < 0.05) higher droplet size (318.91 nm). Therefore, the antimicrobial potential of WFNE3 nanoemulsion in comparison with extract of *W. fruticosa* flower extract was studied against Gram-positive *Staphylococcus aureus*, Gram-negative bacteria *Pseudomonas aeruginosa*, and fungal strain *Candida albicans*, respectively. WFNE3 nanoemulsion sample in comparison to flower extract showed a significantly higher (*p* < 0.05) zone of inhibition against gram-negative bacteria as compared to the control nanoemulsion sample upon storage for 7 days. WFNE3 nanoemulsion sample showed significant (*p* < 0.05) higher inhibition of protein denaturation (57.89%−87.65%) and (55.36%−83.58%) in comparison to control nanoemulsion sample (54.67%−80.28%) and flower extract (51.56%−79.36%), respectively. Due to these biological activities, the WFNE3 nanoemulsion sample could be scaled up to the industrial level for the formulation of varied types of functional foods.

## Introduction

*Woodfordia fruticosa* Kurtz is a well-known plant in the Lythraceae family that grows up to 1,500 m in altitude and is found in India's tropical and subtropical regions. Traditional practitioners have used the plant for a long time, particularly in Southeast Asian countries (1). Although all parts of plants have medical properties, flowers are in high demand in both domestic and international markets. The blossoms are reddish-brown and possess pungent, acrid, cooling, and alexiteric characteristics, acting as a uterine sedative and anthelmintic (2). Sprue, dysentery, intestinal complaint, rheumatism, hematuria, wounds, bleeding, and injuries are all treated with the dried flowers of *W. fruticosa*. Due to their therapeutic characteristics, these flowers are also used in the formulation of fermented Ayurvedic drugs known as “Aristhas and Asavas” (3). Myricetin, oenothein B, isochimacoalin-A, –terpinene, limonene, and oligomers such as woodfordins A, B, C, E, G, H, and I, as well as quercetin, are among the phenolic compounds found in the flowers. The anti-inflammatory activities of quercetin and myricetin are related to their inhibition of the two key enzymes, lipoxygenase and cyclooxygenase of pelargonidin, that play a key role in the pathophysiology of inflammation (1, 4). –terpinene and limonene have antibacterial and antifungal characteristics, and they act by entering the cell membrane and destroying cellular components (5, 6). The consumption of these naturally occurring compounds can reduce the side effects such as edema, heaving, queasiness, diarrhea, coughing, loss of appetite, and abdominal pain caused by synthetic drugs (7). Scientists and various researchers are, therefore, working on the preparation of products from naturally occurring herbs to compensate for the therapeutic gap as well as reactions that are required during the secondary treatment. *W. fruticose* flower due to the presence of phytocompounds has potential health benefits and can be utilized as a remedy for the treatment of various biological disorders. In our previous study, Najda et al. (1), *Woodfordia fruticosa* flower extract was prepared by modified solvent evaporated technique, and its characterization was done using gas chromatography-mass spectroscopy. The compounds identified during characterization were: γ-terpinene, dihydrocarvyl acetate, 1-decalone (cis-trans), cis-7-decen-1-al, tetradecanoic acid, palmitic anhydride, pentadecanoic acid, octadecanoic acid, *n*-hexadecanoic acid, and 3-decyn-1-ol, 2,6-octadien-1-ol, 3,7-dimethyl-, acetate, and €-(geranyl acetate), as well as caryophyllene epoxide, cyclopropaneoctanoic acid, Cyclopropaneoctanoic acid, 2H-1-benzopyran-2-one, 2H-1- benzopyran-2-one, and γ-elemene. All these compounds have potential health benefits, therefore, incorporation of these phytocompounds isolated from the flowers into functional food or as dietary supplements can play a vital role for both consumers and food industries. The isolated compounds are least soluble in water, therefore, are more prone to environmental oxidative stress (8). It is essential to increase the water solubility of these components to achieve substantial floral extract properties. Emulsification is the most effective and common way to improve the functionality of phytocompounds (1). Emulsification has been shown to improve the stability and bioavailability of these components in several *in vitro* and *in vivo* experiments (8). In comparison to plant extract, nanoemulsion has a better antibacterial activity (9). Furthermore, gum arabic is a polysaccharide complex that contains 2% polypeptide and the branches are mainly comprised of 1,3-linked-d-galactopyranosyl units with 1,6-linked -d-galactopyranosyl side chains to which several arabinosyl, uronic acid, and rhamnose residues are attached. In addition, a 43-amino-acid-residue peptide sequence is also present in the native molecular structure of the gum arabic (10). The strong emulsifying characteristics of gum arabic have also been reported due to the existence of inter and intramolecular CH...π interactions (11, 12). The oil phase was desirable for the preparation of *W. fruticosa* flower extract nanoemulsion, hence sunflower oil was selected to disperse the flower extract and to formulate the continuous phase of the nanoemulsion. There are several studies available on the formulation of plant extract-based nanoemulsions; however, no data are available on the formulation of *W. fruticosa* flower extract nanoemulsion. Therefore, the current research work was performed with the following objectives in mind: (i) Preparation and stabilization of *W. fruticosa* extract nano-emulsion; (ii) Characterization of the emulsion; (iii) *in vitro* mineral bioaccessibility of zinc, iron, calcium, and ferritin contents of nanoemulsion using transwell assay; and (iv) Antimicrobial and anti-inflammatory activity of emulsion.

## Materials and methods

### Materials

Flowers *of W. fruticosa* were harvested from the forest of district Mandi located in Himachal Pradesh, India. Analytical reagent grade emulsifying and a surface-active agent such as Gum arabic (spray dried) and tween-80 were procured from Sigma Aldrich Co. St. Louis, MO, USA. From Hi-Media Laboratories Pvt. Ltd., Mumbai, India, ethanol, L-ascorbic acid, calcium chloride, sodium chloride, phosphate buffer, Muller Hinton Agar (MHA), and Sabouraud Dextrose Agar (SDA) were purchased. For the oil phase and dispersion of flower extract, sunflower seed oil was used and it was obtained from CDH Pvt. Ltd., Mumbai, India. For antimicrobial studies, standard pathogenic bacterial strains such as Staphylococcus aureus (MTCC 3160), *Klebsiella pneumoniae* (MTCC 3384), Pseudomonas aeruginosa (MTCC 2295), and *Salmonella typhmurium* (MTCC 1254) and fungal strain of *Candida albicans* (MTCC 183) were obtained from IMTECH, Chandigarh, India.

### Methods

#### Preparation of ethanolic extract

*Woodfordia fruticosa* flowers were picked fresh and to remove dirt and debris, flowers were rinsed with triple distilled water before drying at 30°C in a hot air oven (SGM lab Solutions Private Limited, India). A mechanical grinder was used to ground the dried flowers into a uniform powder (Bajaj, mixer grinder, 900 watts, New Delhi, India). The powdered sample (10 g) was dispersed in 100 ml absolute ethanol (1:10 w/v ratio) in a 250-ml conical flask and placed in an orbital shaker for 72 h (Thermo Fisher Scientific Pvt. Ltd., Mumbai, India). The samples were then filtered using Whatman Filter Paper No. 1 and kept at refrigerator temperature (4–7°C) for a further 72 h. The dried extract was then obtained and stored in an airtight amber-colored glass vial at −20°C for further investigations.

#### Preparation of nanoemulsion

Different concentrations of *W. fruticosa* flower extract (WFE; 0.25, 0.50, 0.75, 1, 1.5, 2, 2.5, 3, 4, and 5%) were dispersed in 10 ml of sunflower (SF) oil using a magnetic stirrer (SPINOT MC 02, Tarsons, Kolkata, India) and the oil phase (WFE + SF) was then mixed in an aqueous phase containing 1% gum arabic and 250 μl tween-80 for 20 min. The final nanoemulsion samples (WFNE0.25-WFNE5) were formulated with the high-energy process using a probe sonicator (Sonics and Materials Inc. New Town, CT, USA) at 5°C, 5.0 s pulse rate, and 25 min (formulation time). The freshly prepared nanoemulsion samples were then kept in air-tight glass containers for further screening (8).

#### Selection of suitable nanoemulsion

##### Creaming index

Creaming stability of freshly prepared nanoemulsion samples was assessed by following the method proposed by Pengon et al. (13) for the selection of suitable nanoemulsion during 15 days of storage. In brief, glass bottles filled with 50 ml nanoemulsions were kept at accelerated temperatures (80°C) and the physical stability of the nanoemulsion samples was then examined by calculating the creaming index.

The following equation was used to determine the creaming index:


$$\begin{array}{l} Creaming\;index\;\left(\%\right)=\frac{VC}{VE}\;\times 100 \end{array}$$

Here, VC is the volume of cream layer after heating; VE is the volume of the emulsified layer.

##### Droplet size and zeta-potential of nanoemulsion

The dynamic light scattering (DLS) technique is an effective method for determining the droplet size of an emulsion system at the nanoscale. All of the emulsion samples (1% v/v) were diluted in deionized water for the droplet size evaluation. Measurements were recorded in triplicates for each sample at 25°C using particle size and zeta potential analyzer (Zetasizer Nano ZS, Malvern Instruments Ltd. Malvern, WR14 1XZ, UK).

#### Influence of processing treatment on droplet size and zeta potential of nanoemulsion

##### Effect of thermal processing

Freshly prepared nanoemulsions were exposed to a series of processing treatments that might come transversely in industrial applications. The effect of all the processing treatments on droplet size and zeta potential was examined.

##### Effect of pH

Hydrogen ion concentration imparts a great influence on the droplet and particle size of the food materials; hence, nanoemulsions were transferred into 50 ml glass beakers and adjusted to different pH ranges (2.0–9.0) using 0.1N NaOH and 0.1N HCl solutions. All the samples were then subjected to droplet size and zeta potential evaluation.

##### Effect of salt concentration

After the storage of the samples for 1 month at a temperature of 30°C, the influence of varied salt concentrations on the droplet size of nanoemulsions was carried out by following the literature (14). In brief, sodium chloride in different concentrations (0.5, 1, and 2 M) was added to confirm the alteration in droplet size and zeta potential.

##### Oxidative stability of nanoemulsion

Oxidative stability of formulated nanoemulsion was examined by determining the TBA values by following the literature (15). Fresh 0.025 M TBA solution was prepared and it was neutralized with 2 M NaOH and citric acid, respectively. TBA reagent was then added to nanoemulsion (5 ml) and was mixed and the mixture was heated instantly in a boiling water bath for 10 min. The sample was then cooled in ice-cold water followed by the addition of cyclohexane (10 ml) and 4 M ammonium sulfate (1 ml). Samples were centrifuged at 5,000 ×g and the orange-red colored solvent was obtained and absorbance was then measured at 532 nm using a UV-visible spectrophotometer (Shimadzu UV-2600i/UV2700i, Tokyo, Japan).

##### *In vitro* antimicrobial assay

The potential antimicrobial activity of nanoemulsion was examined against pathogenic Gram-positive and Gram-negative bacteria viz. *Staphylococcus aureus, Pseudomonas aeruginosa*, and fungi *Candida albicans* using the agar well diffusion method. Herein, 1 × 10^8^ cells/ml bacterial inoculum and 1 × 10^5^ cells/ml fungal inoculum were inoculated on 4% enriched Mueller Hinton agar (MHA) and Sabouraud Dextrose agar plates, respectively. The wells on agar plates were made using a sterile cork borer. The extract was eluted in 8% DMSO and 50 μl of it was transferred into the agar well using a micropipette. The MHA plates containing bacterial inoculum were incubated for 24 h at 37°C and the SDA plate containing fungal inoculum was incubated at 27°C for 72 h. The zone of inhibition diameter was measured using a vernier caliper (12).

##### Time kill study

Time kill study was measured by methods proposed by Majeed et al. (16) Herein, 100 μl of the extracts for each bacterial and fungal sample were taken after a time interval of 0, 18, 24, and 48 h and 0, 24, 48, and 72 h, respectively. Then, samples were diluted serially and spread on plates containing MHA and SDA. The plates were incubated at 37and 72°C, respectively. Log CFU/ml was then calculated after incubation.

##### *In vitro* anti-inflammatory activity

###### HRBC membrane stabilization

Human red blood cell membrane stabilization was performed by the following method proposed by Bains et al. (12). In brief, from a healthy volunteer not administered with non-steroidal anti-inflammatory drugs (NSAID) for 15 days, 5 ml of blood was taken and dissolved in an equal volume of Alsever solution (20.5 g dextrose, 8 g sodium citrate, 0.55 g citric acid, and 4.2 g of sodium chloride dissolved in 1,000 ml distilled water). The mixture was centrifuged at 3,000 × ***g*** for 15 min. After centrifugation, the packed cell thus obtained was washed with iso saline solution. An equal volume of HRBC suspension and extract (500 μl) mg/ml was dissolved in 0.36% hypo saline solution (2 ml) containing phosphate buffer pH 7.4. The mixture is then incubated in biological oxygen demand (BOD) incubator for 30 min at 37°C and after incubation, the suspension was centrifuged at 3,000 ×***g*** for 20 min. Diclofenac sodium and deionized water were used as the positive and negative controls.

Percentage membrane stabilization assay was calculated as:


$$\begin{array}{l} Stabilization\;\left(\%\right)=100-\frac{Ot}{Op}\;\times 100 \end{array}$$

where: Ot is the optical density of the sample and Op is the optical density of the control.

#### Albumin denaturation assay

In this assay, reaction mixture (5 ml) containing fresh egg albumin (200 μl), 2.8 ml buffer solution (pH 6.4), and 2 ml of extracts were mixed and incubated at 37°C at 15 min for 15 min followed by heating up to 70°C for 5 min and absorbance was measured at 660 nm. Deionized water and diclofenac sodium were used as the negative and positive control. Percentage inhibition of protein denaturation was calculated as:


$$\begin{array}{l} Inhibition\;\left(\%\right)=100\;\times \frac{Oa}{Oc}-1 \end{array}$$

where: Oa test sample absorbance and Oc control absorbance (13).

#### Statistical analysis

Statistical analysis of obtained results was carried out by following the method given by Kaushik et al. (17). Statistical differences and standard deviations were calculated by one-way ANOVA (analysis of variance) and Microsoft Excel, respectively. The comparison between the mean calculated by difference value and the standard mean error was determined by a descriptive statistical tool.

## Results and Discussion

### Synthesis of nanoemulsion

**Figure 1:**
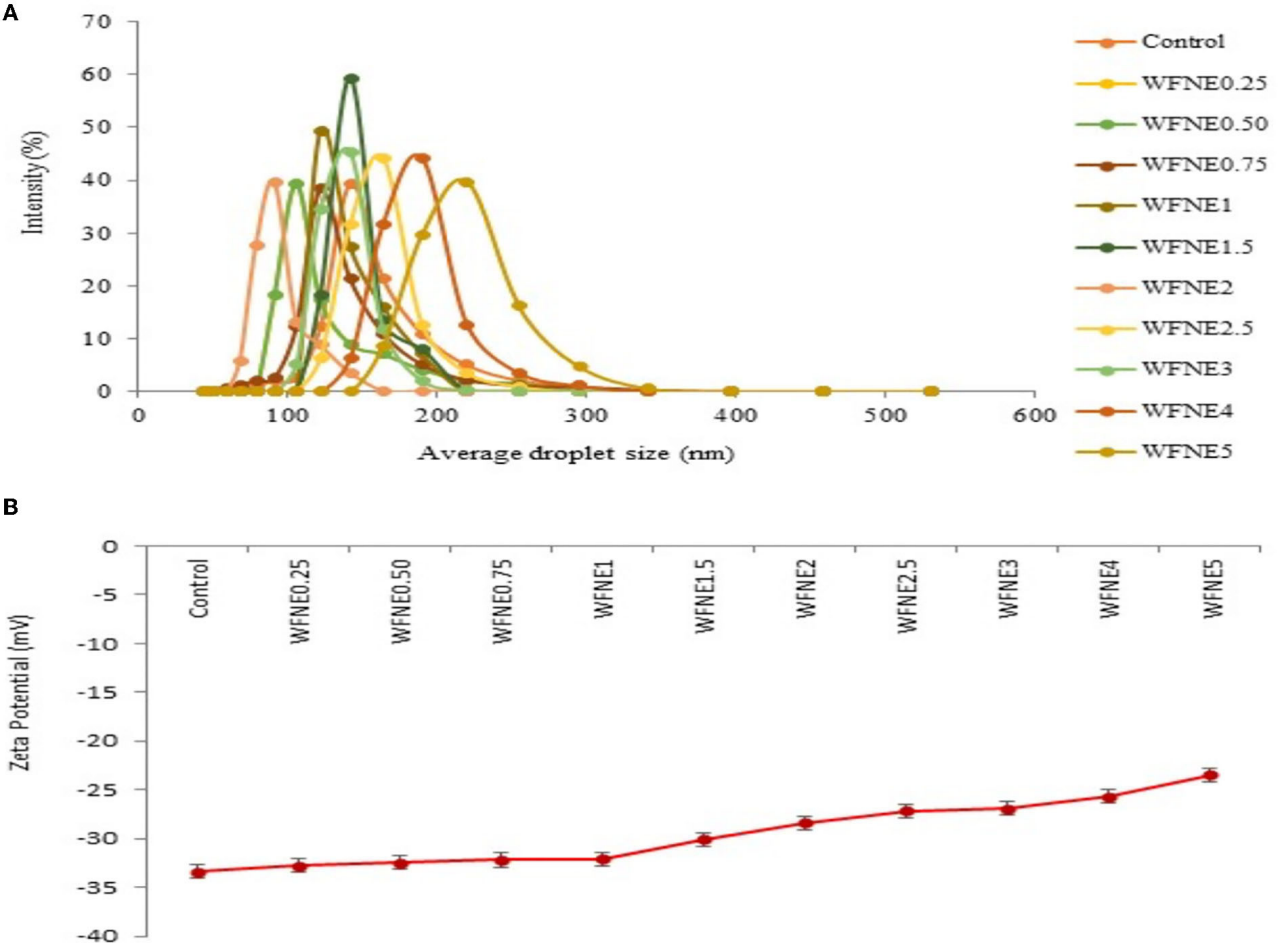
**(A)** Average droplet size of WFNE3 nanoemulsion; **(B)** Zeta potential of WFNE3 nanoemulsion. The error bar represents the standard deviation from the mean values.

The effect of varying concentrations of *W. fruticosa* extract on the formulation of nanoemulsion was initially investigated for droplet size and the results are depicted in Figure 1A. The average droplet size of all the nanoemulsions ranged from 149.25 to 244.33 nm, respectively, and increased droplet size was observed with increasing concentration of extract. Herein, among all the nanoemulsion samples, significantly higher (*p* < 0.05) droplet size was observed for WFNE5; however, samples ranging from WFNE 0.25 to WFNE3 revealed an insignificant (*p* > 0.05) difference. Furthermore, even at a high concentration of flower extract, no visual sign of flocculation or creaming was observed. This emulsion behavior (no visual sign of flocculation) was attained due to a change in the charge distribution of exterior and interfacial viscosity of the emulsion system in the presence of gum arabic and tween-80 (8). The Zeta potential of all emulsion samples was observed and the results are depicted in Figure 1B. Herein, all the samples showed a significant difference in zeta potential and it ranged from −34.4 to −23.5 mV. However, it could be revealed from the results that samples ranging from WFNE0.25 to WFNE1 showed insignificant differences (*p* > 0.05) in zeta potential values of all the emulsion samples, with increasing concentration nanoemulsion samples (WFNE1.5–WFNE5) showed significant difference (*p* < 0.05) in zeta potential. According to the literature, zeta potential characterizes the surface charge and that can be employed for the understanding of physical and thermal stability of nanoemulsion due to electrostatic repulsion. Moreover, zeta potential depends on the composition of emulsion and it is a function of the droplet's environment. Overall, negative charge distribution on formulated nanoemulsion was majorly due to the arrangement of gum arabic molecules with oil phase, surface active agent, and the flower extract through hydrophobic binding moiety. Our results were well supported by the findings of Khan et al. (18) and Yao et al. (19) who observed the electrostatic interaction of emulsifier with continuous phase and surface-active agent with high total charge distribution.

#### Creaming index of nanoemulsion

**Figure 2:**
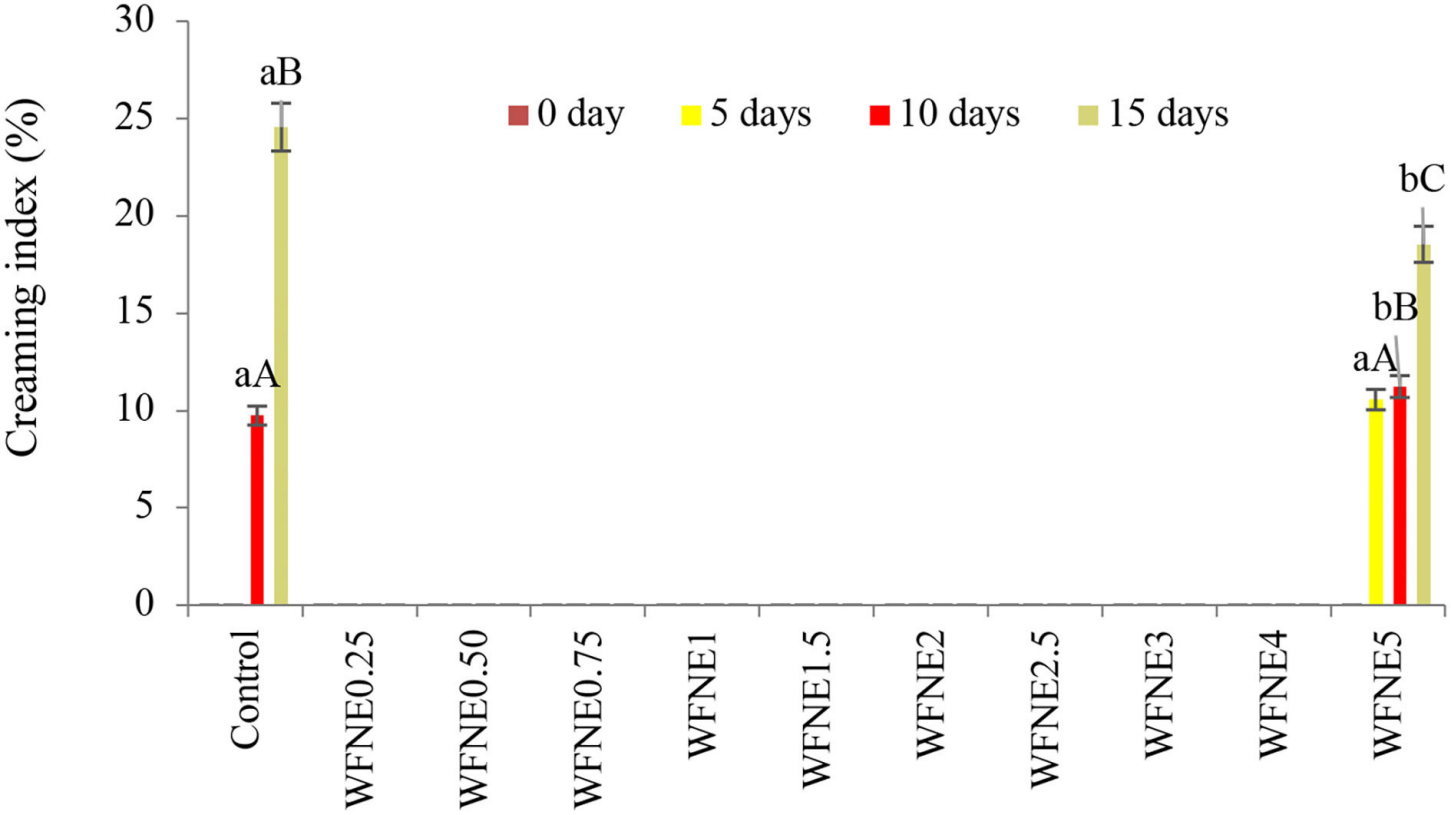
Creaming index of WFNE3 nanoemulsion. The error bar represents the standard deviation from the mean values.

To understand how extract influenced the stability of emulsions, it was necessary to understand the creaming stability processes responsible for destabilizing emulsions in the first place, and the results of creaming stability of control and WFNE0.25–WFNE5 nanoemulsion samples are presented in Figure 2. Herein, the control emulsion sample showed a significantly (*p* < 0.05) higher creaming index (24.56%) in comparison with flower extract nanoemulsion (10.56%−18.54%), and during the 10th day of the storage, visual creaming was observed in the control sample. However, WFNE5 showed a 10.56% to 18.54% creaming index during the 5th, 10th, and 15th days of storage, respectively. The colloidal stability of an emulsion can be controlled by the colloidal interaction forces keeping the droplets apart and evenly dispersed.

Creaming is the mass transfer of emulsion droplets on the surface of the emulsion, driven by the resistance of the droplets of emulsion in the continuous phase. Moreover, the movement of the emulsion droplets can be driven by the density difference between the droplets and the continuous phase, the size of the droplet, and the viscosity of the continuous phase. Moreover, according to Derjaguin–Landau–Verwey–Overbeek theory, the creaming index of colloidal systems potentially depends on the balance of electrostatic repulsive forces and van der Waals attractive forces acting on the interface. Therefore, a theoretical model of nanoemulsion can be anticipated, where the hydrophilic part of the emulsifier projects into the aqueous phase which remains toward molecules of flower extract. Cavitation during the ultra-sonication process exerted chemical distortion of the oil phase. Hence, samples with a lower concentration of *W. fruticosa* were substantially stable in terms of creaming index due to considerably mono-dispersed, small droplet sized, and strong repulsion between extract nanodroplets, adequately coated by gum arabic (8).

#### Thermal stability

**Figure 3:**
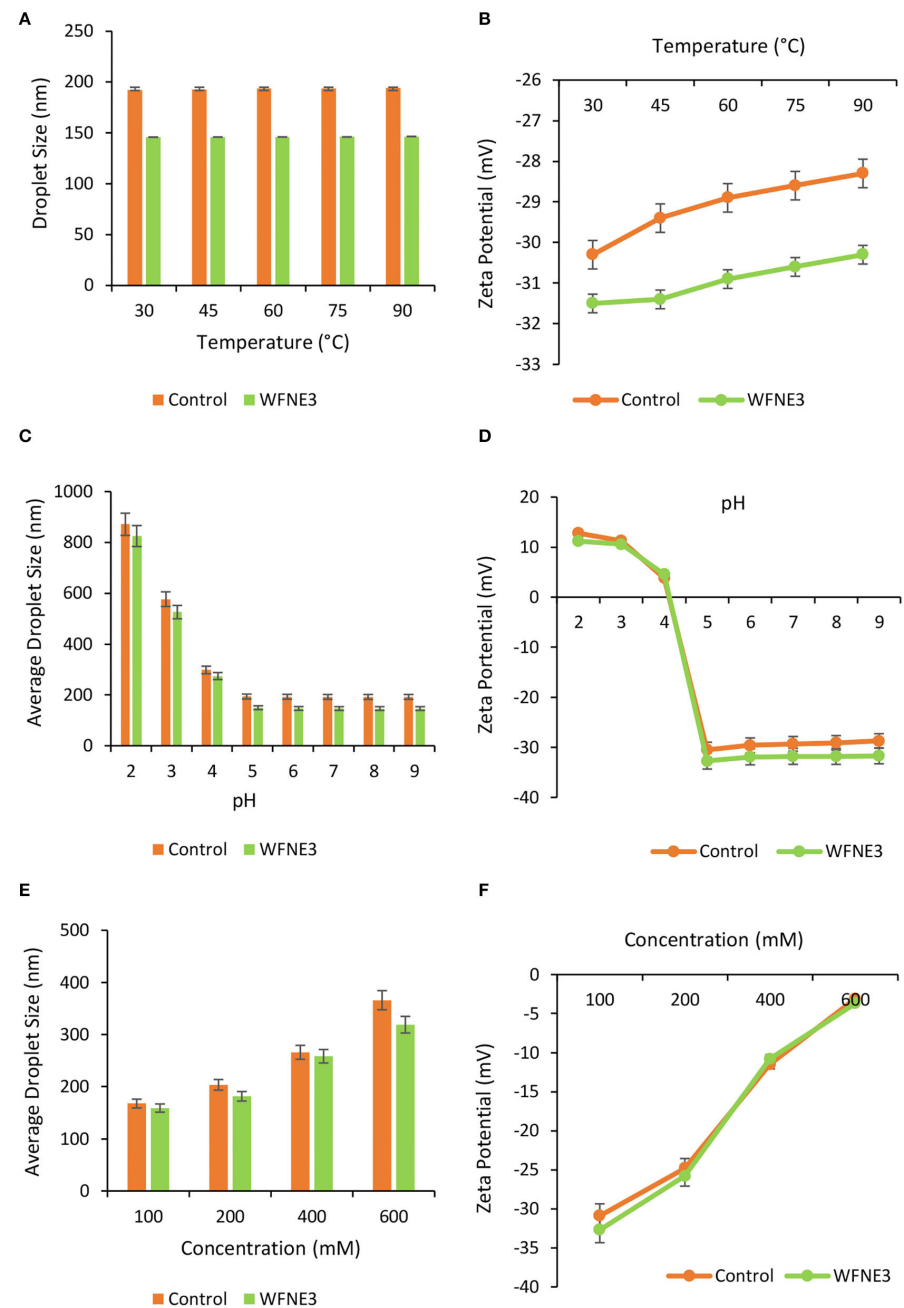
Effect of different ranges of **(A)** temperature on droplet size of WFNE3 nanoemulsion; the **(B)** temperature on zeta potential of WFNE3 nanoemulsion; **(C)** pH on droplet size of WFNE3 nanoemulsion; **(D)** pH on zeta potential of WFNE3 nanoemulsion; **(E)** ionic concentration on droplet size of WFNE3 nanoemulsion; **(F)** ionic concentration on zeta potential of WFNE3 nanoemulsion. The error bar represents the standard deviation from the mean values.

The impact of food processing temperature on food matrixes or packaging components comprising nanoemulsion that reveal the stability during final consumption was examined by illustrating the thermal stability of nanoemulsion [8]. The thermal stability of control and WFNE3 nanoemulsion is depicted in Figures 3A,B and during observations, both WFNE3 and control samples showed significant (*p* < 0.05) differences from each other. Herein, as compared to the control nanoemulsion (194.22 nm), the WFNE3 (146.56 nm) nanoemulsion showed significantly (*p* < 0.05) higher thermal stability when it was correlated with average droplet size. Moreover, with an increasing temperature rate, an insignificant (*p* > 0.05) difference was observed in the average droplet size of the nanoemulsion samples. During the thermal processing of the selected WFNE3 nanoemulsion, no visual creaming or coalescence was observed. On the other hand, in the case of charge distribution of the colloidal system, with the increase in temperature, an insignificant difference in the zeta potential of WFNE3 (−30.3 mV) was observed. This could be due to the developed steric repulsion by surface-active agent and emulsifier and it was not extensively adequate due to the creation of thin interfacial layers. Therefore, as a result, the droplets of the colloidal system were highly stable against aggregation. As well, interfacial colloidal surface tension in selected WFNE3 nanoemulsion was interrelated to the stimulus of Laplace pressure, in which lower values enable the rupture of the droplets, creating minor droplet sizes and higher stability against aggregation or coalescence with increasing temperature (20).

#### pH stability

The impact of variation in pH upon the droplet size and charge distribution (zeta potential) of the control and WFNE3 nanoemulsion was examined and results are presented in Figures 3C,D. Herein, at varied pH ranges, WFNE3 showed significantly higher (*p* < 0.05) stability in comparison with the control nanoemulsion sample. The colloidal system of the WFNE3 nanoemulsion sample at pH 5–9 showed significantly lower (*p* < 0.05) average droplet size as compared to pH ranging from 2 to 4, and it was found to be highly stable coalescence and Ostwald ripening. However, at pH 4, a thin layer of lipid droplets on the surface of the WFNE3 nanoemulsion with a lower droplet size (290.27 nm) was observed. Despite the small droplet size, there was calescence in the sample. The WFNE3 nanoemulsion sample was highly unstable to phase separation at pH 2 and 3. This result was possibly due to the revelation of amino chains of emulsifier molecules with an amplified rate of protonation, triggered by the demulsification (21).

#### Ionic strength

Different salt concentrations were induced to the colloidal system of the selected nanoemulsion and the influence on droplet size and zeta potential was evaluated (Figures 3E,F). Herein, WFNE 3 nanoemulsion sample showed significantly (*p* < 0.05) higher stability in terms of ionic strength in comparison with the colloidal system of the control nanoemulsion sample. However, with an increasing concentration of salt, the colloidal system of the WFNE3 sample showed a significantly (*p* < 0.05) higher droplet size (318.91 nm). This result was obtained due to structural chemistry and polyanionic charge distribution on the surface of gum arabic which resulted in coulombic repulsion; however, negatively charged carboxylic moiety led to electrostatic interaction between positive and negative charges of sodium ion which increased in droplet size. Furthermore, the zeta potential of the colloidal system of WFNE3 nanoemulsion was greatly influenced by the electrostatic interaction and a significant decrease (*p* < 0.05) in the overall charge distribution of the nanoemulsion sample was observed. The presence of positive charge on amino acid groups of gum arabic is bound with negatively charged chloride ions (17). Apart from the increase in droplet size of nanoemulsion, the colloidal stability of nanoemulsion did not affect due to increased salt concentration, hence no visual coalescence and oiling off were observed.

#### TBA value

**Figure 4:**
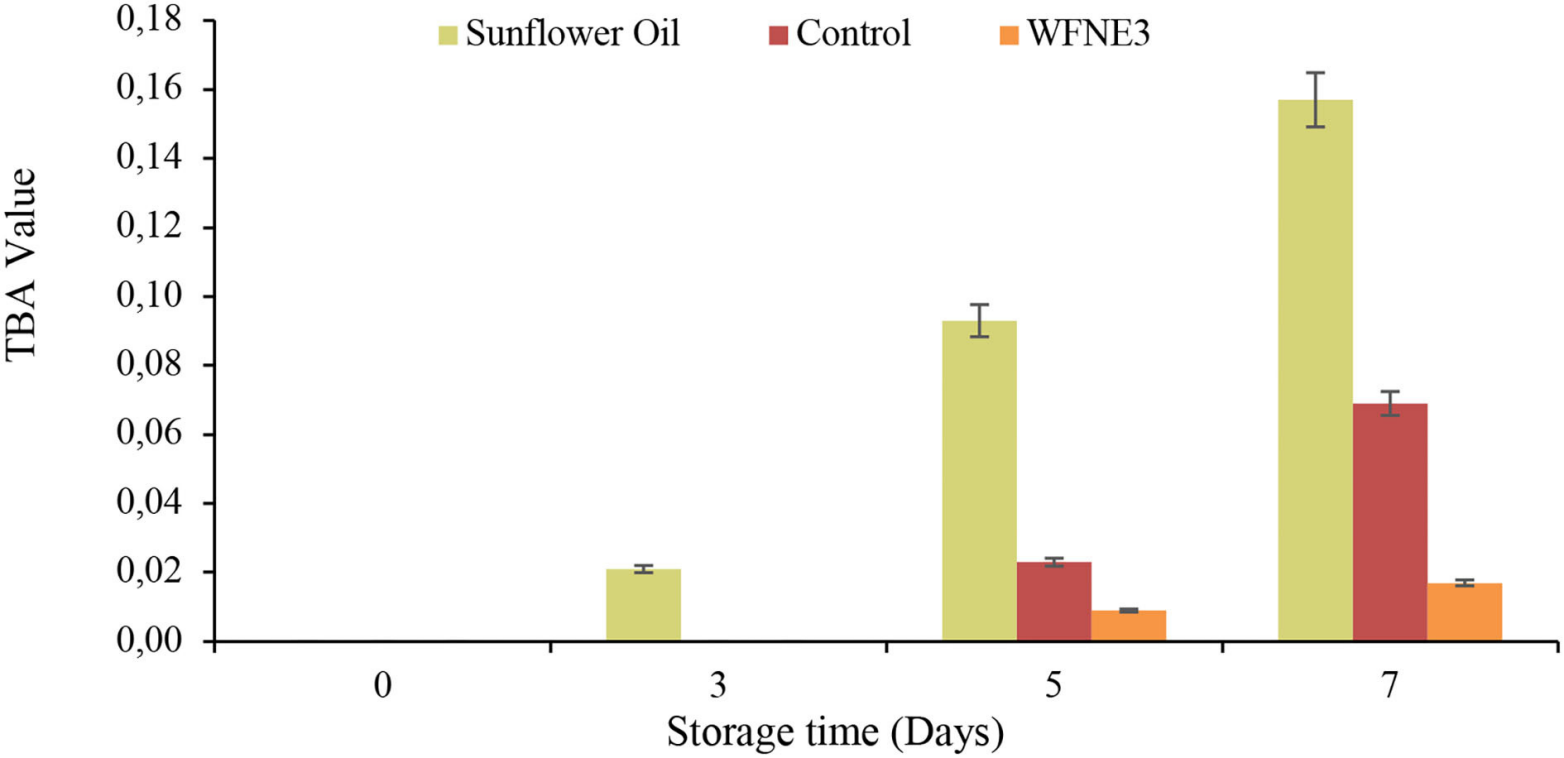
Effect of storage on TBA value of WFNE3 nanoemulsion. The error bar represents the standard deviation from the mean values.

Oxidative stability was measured for both of the nanoemulsion samples and the results of TBA value in comparison with sunflower oil are depicted in Figure 4. Varied range and significant (*p* < 0.05) difference of oxidative stability in terms of TBA value for sunflower oil (0.00–0.157), control nanoemulsion, and WFNE3 nanoemulsion (0.00–0.017) was observed during the storage. However, the control emulsion sample and WFNE3 emulsion sample revealed in-significant differences (*p* > 0.05) with each other, whereas, a significant (*p* < 0.05) difference was observed in the TBA value of sunflower oil and nanoemulsion samples during the 3rd, 5th, and 7th day of storage, respectively. The higher oxidative stability of the colloidal system of WFNE3 nanoemulsion was due to the occurrence of *W. fruticosa* flower extract (22) and our results were well supported by the findings of Chawla et al. (8) who observed higher oxidative stability in terms of TBA value for gum arabic-stabilized *Rhododendron arboreum* flower extract nanoemulsion.

#### Antimicrobial activity

**Figure 5:**
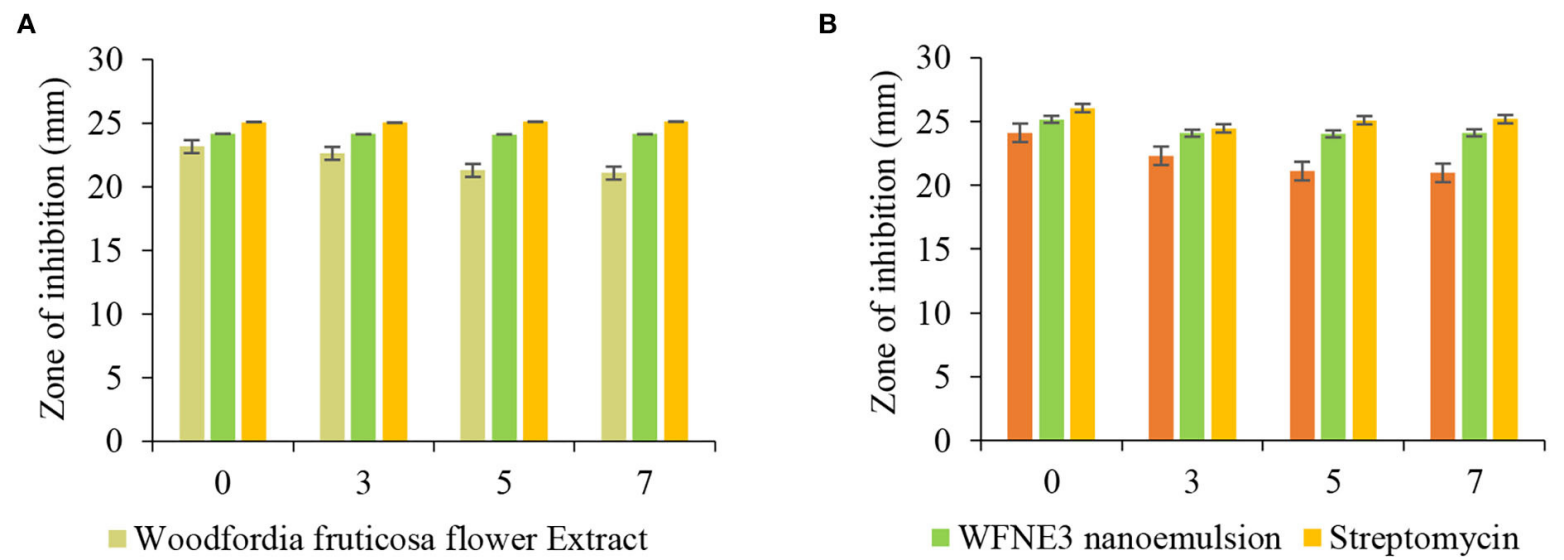
Effect of storage on the antimicrobial efficacy of WFNE3 nanoemulsion against (**A)** *Pseudomonas aeruginosa* and *Staphylococcus aureus;* and (**B)** *Candida albicans*. The error bar represents the standard deviation from the mean values.

The antimicrobial potential of WFNE3 nanoemulsion in comparison with extract of *W. fruticosa* flower extract was assessed against pathogenic gram-positive and gram-negative bacterial and fungal strains. The results were represented in Figures 5A,B. Herein, the flower extract and nanoemulsion were stored at 37°C for 7 days and then evaluated for their antimicrobial activity. In the present study, it has been observed that stored WFNE3 nanoemulsion samples in comparison to flower extract showed a significantly higher (*p* < 0.05) zone of inhibition against gram-negative bacteria. However, in comparison to positive control in terms of zone of inhibition, there observed a non-significant difference. Furthermore, the flower extract showed significantly (*p* < 0.05) less antimicrobial activity against gram-negative bacteria *P. aeruginosa* during the 7th day of storage, and the zone of inhibition decreased from 227.76±0.1 mm to 17.34±0.05 mm; however, on the 5^th^ and 7^th^ day of storage, non-significant difference (*p* < 0.05) in antimicrobial activity was observed. During storage in the case of WFE nanoemulsion, no decrease was observed in the zone of inhibition. In comparison to flower extract, WFNE3 is highly susceptible to gram-positive bacteria and fungus and showed a significantly higher (*p* < 0.05) zone of inhibition against them. Also, during the 7th day of storage, WFNE3 nanoemulsion showed high antimicrobial susceptibility as compared to the control nanoemulsion sample and have a non-significant (*p* < 0.05) difference in the zone of inhibition. There was a significant decrease (*p* < 0.05) in the zone of inhibition for both bacterial and fungal strains shown by flower extract during storage, hence it was concluded from the results that the antimicrobial susceptibility of flower extract decreases when stored at 37°C for 7 days while WFNE3 showed promising results. Here, the WFNE3 extract was least susceptible to gram-negative bacteria *P. aeruginosa* in comparison to *S. aureus* and *Candida albicans*. In the case of gram-positive bacteria *S. aureus*, the cell wall is composed of a thick hydrophobic cell wall that permits the passage of bioactive components to pass through the cell membrane (23), while in the case of gram-negative bacteria, the cell wall is composed of lipopolysaccharides that cause the difference in hydrophobic properties and induce mutation in porins and other factors that result in resistance against several extracts, nanoemulsion, and synthetic antibiotics. The WFNE3 nanoemulsion showed remarkable antifungal activity against *C. albicans* with a zone of inhibition of 25.62 mm. The bioactive compounds present in the sample diffuse resulting in the disruption of the cell membrane of fungal strain. Another reason for remarkable antimicrobial activity is that nanoemulsion due to their nano size results in an increase in the contact surface of the sample with microorganisms, thereby resulting in an improvement of accessibility of bioactive compounds and their ability to penetrate inside the cell membrane (24).

### Time kill study

**Figure 6:**
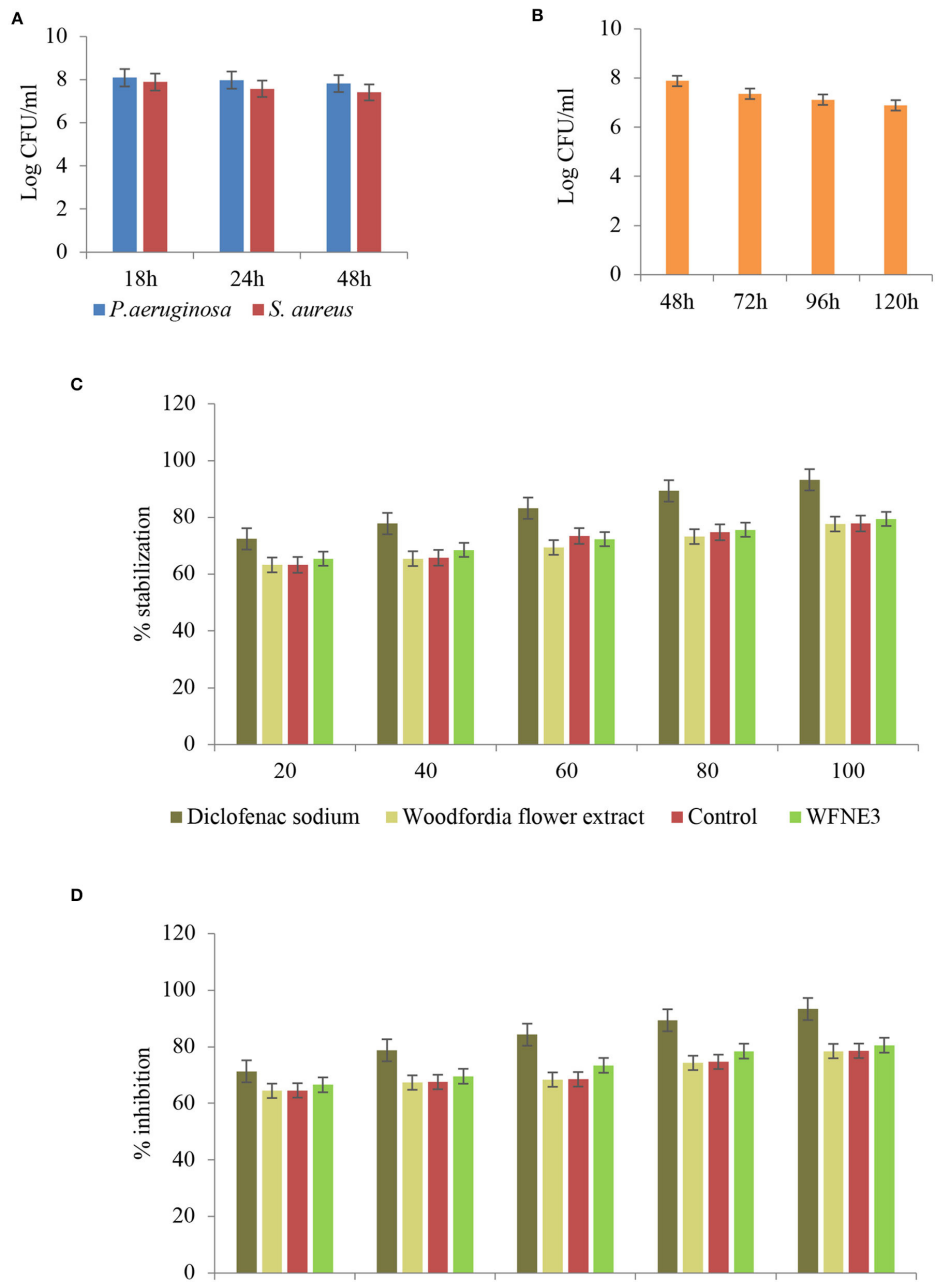
Time kill kinetics of WFNE3 nanoemulsion against (**A**) *S. aureus* and *P. aeruginosa*, (**B**) *C. albicans*, (**C**) Anti-inflammatory activity of WFNE3 nanoemulsion with HRBC membrane stabilization, and (**D)** Anti-inflammatory activity of WFNE3 nanoemulsion using BSA denaturation assay.

Time kill study of the WFNE3 nanoemulsion sample was performed and results are presented in Figures 6A,B. In the present study with the increase in time interlude, nanoemulsion WFNE3 showed less significance (*p* < 0.05; 7.82 and 7.76 log CFU/ml) in comparison to control nanoemulsion (7.90 and 7.82 log CFU/ml) and flower extract (7.98 and 8.02 log CFU/ml) for both bacterial strains. The inhibitory effect of the WFNE3 nanoemulsion sample on *S. aureus* is due to the bioactive compounds present in it. These bioactive compounds suppose to inhibit the synthesis of protein and biochemical pathways and result in the destruction of cell membrane structure. The gram-negative bacterial strain *P. aeruginosa* showed a higher log CFU/ml value, this is due to the reason that in gram-negative bacteria, the outer cell membrane consists of a layer of a phospholipid bilayer and lipopolysaccharides that are embedded with porin proteins and β-barrel channels. This structure prevents the penetration of antibiotics and other active components to enter the cells (25). Another foremost reason for the resistance of gram-negative bacteria is the development of efflux pumps that expel the components toxic to the cell and the production of enzymes that act either by breaking or causing alteration in the antibiotic structure and bioactive components by using mutational changes or genes acquisition (26). Similarly, in the case of *C. albicans*, nanoemulsion showed significantly lower (*p* < 0.05) 7.72 log CFU/ml values as compared to control nanoemulsion (7.80 log CFU/ml) and flower extract (7.98 CFU/ml). The reduction in log CFU/ml value is due to the reason that yeast lacks surface-active hydrophobin proteins and has hydrophilic nature; therefore, the hydrophobic antifungal components can readily be accumulated at the conidia of fungal strains (1, 27).

#### Anti-inflammatory activity

Albumin denaturation assay and HRBC membrane stabilization assay were used for the anti-inflammatory assay and the results are depicted in Figure 6C. In the present study, a significant (*p* < 0.05) difference was observed in the anti-inflammatory activity of flower extract, WFNE3, and the control nanoemulsion sample. WFNE3 nanoemulsion, control nanoemulsion sample, as well as flower extract showed inhibition of protein and stabilization of HRBC membrane in a dose-dependent manner (20–100 μg/ml). WFNE3 nanoemulsion sample showed significant (*p* < 0.05) higher inhibition of protein denaturation (55.36%−83.58%) in comparison to control nanoemulsion sample (54.67%−80.28%) and flower extract (51.56%−79.36%), respectively. However, in comparison to WFNE3 nanoemulsion, control nanoemulsion and flower extract standard Diclofenac sodium salt showed significantly higher (*p* < 0.05) percentage protein denaturation inhibition and HRBC membrane stabilization. HRBC membrane is similar to the lysosomal membrane; therefore, the assay is considered for limiting inflammation response (28, 29). In addition to this, the anti-inflammatory response in the case of inflammatory disease is well correlated with the denaturation of proteins. Tissue injuries result in the denaturation of proteins that are constituted by cells and intracellular components. The emulsification of flower extract improves the functionality of flower extract which is supposed to inhibit the synthesis or release of inflammatory mediators as well as stabilization of cell membrane (5). Also, the effective anti-inflammatory activity of flower extract results due to the synergistic effect of both the sunflower oil and droplet size in a continuous phase of the emulsion.

## Conclusion

The less bioactivity and release of undesirable metabolites from plant extract results due to environmental stress. To overcome this problem, the formulation of gum arabic-stabilized *W. fruticosa* nanoemulsion was formulated to enhance the bioactivity and functionality of phenolic compounds, which showed a strong health-promoting effect. Among the samples with various additives of *W. fruticosa* extract, emulsions with 3% addition showed the best properties during the stability test, antimicrobial, anti-inflammatory, and antioxidant activities. It was observed that the WFNE3 nanoemulsion sample also showed higher stability to creaming or flocculation over a wide range of temperature, pH, and salt concentrations. Also, during storage, the stability of sunflower oil and flower extract toward oxidation is improved by nanoemulsion. In comparison to flower extract, the nanoemulsion sample showed effective antimicrobial activity against standard bacterial and fungal strains. The WFNE3 also showed significantly (*p* < 0.05) enhanced anti-inflammatory activity and antioxidant activity. Therefore, in the present study, it is determined that gum arabic-stabilized nanoemulsion showed remarkable antimicrobial, antioxidant, and anti-inflammatory activities and hence can be used as an effective food preservative.

## Data availability statement

The original contributions presented in the study are included in the article/supplementary material, further inquiries can be directed to the corresponding authors.

## Author contributions

AN, PC, and AB: conceptualization, formal analysis, investigation, and writing original draft preparation. AN and PC: methodology. PC and AB: software validation. AB: resources. JK and PC: data curation, visualization, and supervision. AN, JK, and PC: review and editing. All authors contributed to the article and approved the submitted version.

## Funding

The support by the Department of Food Technology and Nutrition, Lovely Professional University is gratefully acknowledged. The research project was financially supported by the Minister of Education and Science under the program entitled Regional Initiative of Excellence for the years 2019–2023, Project No. 010/RID/2018/19, and the amount of funding was 12.000.000 PLN.

## Conflict of interest

The authors declare that the research was conducted in the absence of any commercial or financial relationships that could be construed as a potential conflict of interest. The handling editor KS declared a past co-authorship with the author PC.

## Publisher's note

All claims expressed in this article are solely those of the authors and do not necessarily represent those of their affiliated organizations, or those of the publisher, the editors and the reviewers. Any product that may be evaluated in this article, or claim that may be made by its manufacturer, is not guaranteed or endorsed by the publisher.

## Abbreviations

DLS: dynamic light scattering
DPPH: 2, 2-diphenyl-1-picrylhydrazyl
RRE: red rice extract
GA: Gum arabic
TBA: 2-thiobarbituric acid
BSA: bovine serum albumin.

## References

[1] Najda A Bains A Chawla P Kumar A Balant S Walasek-Janusz M. Assessment of anti-inflammatory and antimicrobial potential of ethanolic extract of *Woodfordia fruticosa* flowers: GC-MS analysis. Molecules. (2021) 26:7193. 10.3390/molecules26237193 34885782 PMC8659256

[2] Dubey D Patnaik R Ghosh G Padhy RN. *In vitro* antibacterial activity, GC-MS analysis of *Woodfordia fruticosa* Kurz leaf extract and host toxicity testing with *in vitro* grown lymphocytes from human umbilical cord blood. Osong Publ Health Res Persp. (2014) 5:298–312. 10.1016/j.phrp.2014.08.001 25389517 PMC4225590

[3] Arya A Al-Obaidi MMJ Karim RB Taha H Khan AK Shahid N. Extract of *Woodfordia fruticosa* flowers ameliorates hyperglycemia, oxidative stress and improves β-cell function in streptozotocin–nicotinamide induced diabetic rats. J Ethnopharmacol. (2015) 175:229–40. 10.1016/j.jep.2015.08.057 26342523

[4] Mishra S Sonter S Dwivedi MK Singh PK. Anti sickling potential and chemical profiling of traditionally used *Woodfordia fruticosa* (L.) Kurz leaves. Arab J Chem. (2022) 15:103539. 10.1016/j.arabjc.2021.103539

[5] Malik A Najda A Bains A Nurzyńska-Wierdak R Chawla P. Characterization of citrusnobilis peel methanolic extract for antioxidant, antimicrobial, and anti-inflammatory activity. Molecules. (2021) 26:4310. 10.3390/molecules26144310 34299584 PMC8306028

[6] Shubha JR Bhatt P. Functional attributes of polyphenol-rich *Woodfordia fruticosa* extract: an active ingredient in traditional Indian medicine with nutraceutical potential. J Herb Med. (2021) 29:100488. 10.1016/j.hermed.2021.100488

[7] Kumar MH Prabhu K Rao MRK Sundaram RL Shil S Kumar SA. The GC MS study of one Ayurvedic medicine, Vasakadyaristam. Res J Pharm Technol. (2019) 12:569–73. 10.5958/0974-360X.2019.00101.X

[8] Chawla P Kumar N Kaushik R Dhull SB. Synthesis, characterization and cellular mineral absorption of nanoemulsions of *Rhododendron arboreum* flower extracts stabilized with gum arabic. J Food Sci Technol. (2019) 56:5194–203. 10.1007/s13197-019-03988-z 31749466 PMC6838407

[9] Ghazy OA Fouad MT Saleh HH Kholif AE Morsy TA. Ultrasound-assisted preparation of anise extract nanoemulsion and its bioactivity against different pathogenic bacteria. Food Chem. (2021) 341:128259. 10.1016/j.foodchem.2020.128259 33068847

[10] Mejia Tamayo V Nigen M Apolinar-Valiente R Doco T Williams P Renard D. Flexibility and hydration of amphiphilic hyperbranched arabinogalactan-protein from plant exudate: a volumetric perspective. Colloids and Interfaces. (2018) 2:11. 10.3390/colloids2010011

[11] Sethuraman S. Rajendran, KIs gum arabic a good emulsifier due to CH... π interactions? How urea effectively destabilizes the hydrophobic CH. π interactions in the proteins of gum arabic than amides and GuHCl? ACS Omega. (2019) 4:16418–28. 10.1021/acsomega.9b01980 31616820 PMC6787882

[12] Bains A Chawla P Tripathi A Sadh PKA. comparative study of antimicrobial and anti-inflammatory efficiency of modified solvent evaporated and vacuum oven dried bioactive components of *Pleurotus floridanus*. J Food Sci Technol. (2021) 58:3328–37. 10.1007/s13197-020-04891-8 34366450 PMC8292549

[13] Pengon S Chinatangkul N Limmatvapirat C Limmatvapirat S. Development of antimicrobial nanoemulsions containing *Nelumbo nucifera* extract. Key Eng Mater. (2020) 859:226–31. 10.4028/www.scientific.net/KEM.859.226

[14] Li J Hwang IC Chen X Park HJ. Effects of chitosan coating on curcumin loaded nano-emulsion: study on stability and *in vitro* digestibility. Food Hydrocoll. (2016) 60:138–47. 10.1016/j.foodhyd.2016.03.016

[15] Sharma A Shree BS Arora S Kapila S. Preparation of lactose-iron complex and its cyto-toxicity, *in-vitro* digestion and bioaccessibility in Caco-2 cell model system. Food Bioscience. (2017) 20:125–30. 10.1016/j.fbio.2017.10.001

[16] Majeed H Liu F Hategekimana J Sharif HR Qi J Ali B. Bactericidal action mechanism of negatively charged food grade clove oil nanoemulsions. Food Chem. (2016) 197:75–83. 10.1016/j.foodchem.2015.10.015 26616926

[17] Kaushik R Chawla P Kumar N Janghu S Lohan A. Effect of premilling treatments on wheat gluten extraction and noodle quality. Food Sci Technol Int. (2018) 24:627–36. 10.1177/1082013218782368 29911411

[18] Khan BA Akhtar N Khan H Braga VDA. Development, characterization and antioxidant activity of polysorbate based O/W emulsion containing polyphenols derived from *Hippophae rhamnoides* and *Cassia fistula*. Braz J Pharm Sci. (2013) 49:763–73. 10.1590/S1984-82502013000400016

[19] Yao X Nie K Chen Y Jiang F Kuang Y Yan H. The influence of non-ionic surfactant on lipid digestion of gum Arabic stabilized oil-in-water emulsion. Food Hydrocoll. (2018) 74:78–86. 10.1016/j.foodhyd.2017.07.043

[20] Nazarzadeh E Sajjadi S. Thermal effects in nanoemulsification by ultrasound. Ind Eng Chem Res. (2013) 52:9683–9. 10.1021/ie4003014

[21] Galvão KCS Vicente AA Sobral PDA. Development, characterization, and stability of O/W pepper nanoemulsions produced by high-pressure homogenization. Food Bioprocess Technol. (2018) 11:355–67. 10.1007/s11947-017-2016-y

[22] Gupta A Eral HB Hatton TA Doyle PS. Nanoemulsions: formation, properties and applications. Soft Matter. (2016) 12:2826–41. 10.1039/C5SM02958A 26924445

[23] Gong H Zhang J Hu X Li Z Fa K Liu H. Hydrophobic control of the bioactivity and cytotoxicity of de novo-designed antimicrobial peptides. ACS Appl Mater Interfaces. (2019) 11:34609–20. 10.1021/acsami.9b10028 31448889

[24] Da G Silva C de Oliveira Filho JG De Mori M Ribeiro M Oliveira de Souza CW. Antibacterial activity of nanoemulsions based on essential oils compounds against species of Xanthomonas that cause citrus canker. Biointerface Res Appl Chem. 2022, 12, 1835–45. 10.33263/BRIAC122.18351846

[25] Richter MF Drown BS Riley AP Garcia A Shirai T Svec RL. Predictive compound accumulation rules yield a broad-spectrum antibiotic. Nature. (2017) 545:299–304. 10.1038/nature22308 28489819 PMC5737020

[26] Venter H. Reversing resistance to counter antimicrobial resistance in the World Health Organisation's critical priority of most dangerous pathogens. Biosci Rep. (2019) 39:BSR20180474. 10.1042/BSR20180474 30910848 PMC6465202

[27] Paraszkiewicz K Moryl M Płaza G Bhagat D Satpute SK Bernat P. Surfactants of microbial origin as antibiofilm agents. Int J Environ Health Res. (2021) 31:401–20. 10.1080/09603123.2019.1664729 31509014

[28] Boniface PK Verma S Shukla A Khan F Srivastava SK Pal A. Membrane stabilisation: a possible anti-inflammatory mechanism for the extracts and compounds from *Spathodea campanulata*. Nat Prod Res. (2014) 28:2203–7. 10.1080/14786419.2014.930858 25145995

[29] Sharma S Kota K Ragavendhra P. HRBC membrane stabilization as a study tool to explore the anti inflammatory activity of *Alliumcepa* Linn. –Relevance for 3R. J Adv Med Dent Sci Res. (2018) 6:30–4. 10.21276/jamdsr

